# Chronic Rheumatic Carditis; Senecio Aureus as a Remedy in the Rheumatic Diathesis, Etc.

**Published:** 1879-09

**Authors:** N. S. Davis

**Affiliations:** Chicago


### CHICAGO MEDICAL JOURNAL & EXAMINER.

VOL. XXXIX.—SEPTEMBER, 1879.—NO. 3.

[In these pages dimension and weight are expressed in terms of the Metric System, and temperature in degrees of the Centigrade Scale.]

### Original Lectures.

ARTICLE I.

### CHRONIC RHEUMATIC CARDITIS; SENECIO AUREUS AS A REMEDY IN THE RHEUMATIC DIATHESIS, ETC. Clinical Lecture delivered in the Medical Wards of the Mercy Hospital, June 13, 1879. By N. S. DAVIS, M.D., of Chicago.

*Gentlemen of the class:* On this, the last clinical day of the present college term, I bring to your attention a case which will fairly represent a class of cases sufficiently numerous and important to claim your careful study.

This man, aged about 50 years, a resident of the interior of the state, by occupation a farmer, and presenting the outward appearance of good physical development and fair nutrition, was admitted to the hospital yesterday, and gave the following history and symptoms: Had enjoyed good health until about five years since, when after severe exposure to cold and wet he was attacked with sub-acute articular rheumatism. From this he apparently recovered in a few weeks and resumed his ordinary work, but has ever since been subject, especially during the cold and wet seasons of the year, to more or less rheumatic pains, more generally in the back, hips and left side of the chest. During the last year, he says, he has felt almost constantly, a dull, heavy, uncomfortable feeling below the left nipple, and sometimes extending to the left shoulder and arm ; with shortness of breath and excited action of the heart on walking quick or going up stairs. It is chiefly on account of the continuance and rather steady increase of these latter symptoms, that he has sought admission to the hospital for treatment. He has some degree of constipation, and much of the time his food is slow in digesting, giving rise to gaseous eructations and sometimes vomiting. The urinary secretion is nearly natural, though sometimes scanty and highly colored. Percussion reveals nothing abnormal in the chest, unless it be a very slightly increased area of the cardiac dulness. Neither does auscultation afford any morbid sounds connected with respiration, but if each of you will in turn take the stethoscope and listen over the cardiac region, you will hear a well marked, moderately rough sound immediately following the systole or contraction of the ventricles, and rendering the interval between the first and second sounds of the heart indistinct. If you adjust the funnel of your stethoscope to different parts of the cardiac space, you will find the morbid sound or bellows murmur most loud and distinct about one inch in a direct line below the left nipple, which is nearly over the apex of the heart. This, with the fact that the sound follows the systole, indicates the mitral valve or left auriculo-ventricular opening as the special place of obstruction. While listening to the cardiac sounds, you also become conscious that the impulse of the heart is greater than natural, because it imparts undue motion to the walls of the chest. By this increased force of impulse, coupled with some increase in the area of cardiac dullness before mentioned, it is evident that there is moderate hypertrophy of the muscular structures, as well as thickening of the mitral valve, in this case. It is equally evident, from the history of the patient, that these pathological conditions have originated from rheumatic inflammation involving the endocardial membrane of the left cavities of the heart, probably commencing with the first attack of general rheumatic fever four or five years since. It may have escaped attention at that time, and so far disappeared with the subsidence of that general attack as to leave only slight thickening and irritability of the cardiac valve, yet each subsequent renewal of rheumatic trouble has increased this, until the cardiac changes have now become well marked and permanent. Such cases as this, strikingly illustrate the importance of the injunction I urged upon you so emphatically when lecturing on the general subject of rheumatism, namely, that the physician while attending any case of acute or subacute rheumatism, should note the condition of the heart by careful auscultation at every visit. He should do so, not merely because the cardiac structures become so frequently involved, but because early detection, followed by judicious and persistent treatment, will generally prevent those changes, that if neglected are almost certain to become permanent, and, sooner or later, disastrous to the patient.

*Pathology:* You will best understand the nature and tendency of the cardiac changes in this and similar cases, if you recall what has already been taught you in regard to the nature and effects of rheumatic inflammation in other structures. The exudations in rheumatic inflammation are pre-eminently plastic, leading readily to thickening and induration of the fibrous or connective tissues in any part of the body. The thickened and hardened condition of the ligaments of the smaller joints in the hands and feet, resulting from persistent or frequently recurring rheumatic attacks, is familiar to you all.

So when the endocardial membrane lining the left cavities of the heart, of which the mitral valve is a part, becomes the seat of rheumatic inflammation, strictly parallel changes result. If the inflammation assumes a chronic form, or frequently recurs, the consequent thickening and hardening of the membrane, especially that part of it entering into the valve, becomes permanent, and necessarily presents more or less obstruction to the easy flow of the blood from the left auricle into the ventricle.

It is this obstruction, presented by the altered state of the valve, that causes the rough or bellows murmur you have heard just after the impulse or systole, in the case before you. The obstruction thus established between the left auricle and ventricle constantly tends to keep the auricle over-full of blood, and the ventricle correspondingly deficient in its supply. In the early part of the history of such cases, while the obstruction is slight, the patient feels but little inconvenience while quiet and the heart is acting slowly, because under such circumstances the ventricle receives a fair supply of blood between each systolic contraction. But when by active exercise, exertion, or mental excitement these contractions are made more frequent, the walls of the ventricle contract on an inadequate supply of blood, and not only the auricle, but the pulmonary veins also, become over full and the breathing oppressed. A little reflection will show you that such a state, continuing through months and years, would almost necessarily induce a progressively increasing concentric hypertrophy of the walls of the left ventricle, with diminution of its capacity, and corresponding increase of capacity and fullness of the left auricle and pulmonary veins, with more or less constant shortness of breath and sense of oppression in the chest. When this stage has arrived, and the movable volume of air received into the air cells of the lungs is diminished by the habitual pressure of the over-full pulmonary capillaries, the blood itself becomes imperfectly oxygenated and decarbonized, and consequently cannot afford the natural degree of stimulus, either to nervous sensibility or secretory action. From this come feelings of langour, lassitude and indisposition to active physical or mental exertion, on the one hand; and on the other, indigestion from deficient gastric secretion, constipation of the bowels, and scantiness of urine. Finally, under the continuance and steady progress of these influences, the patient's breathing becomes so oppressed that he cannot assume the recumbent position at night; the heart's action becomes unsteady or intermitting; the digestive organs much disordered; the urinary secretion very scanty, and often albuminous; the general capillary circulation enfeebled, with general tendency to œdema, especially of the lower extremities or whatever parts are most dependent. Having reached this stage, the further progress of the case places the patient in an extremely distressing condition. He is constantly weary and desirous of sleep, yet so oppressed for breath that the sense of suffocation arouses him every few moments, both night and day; the urine becomes so nearly suppressed that nervous twitchings and other symptoms of uremic poisoning are often present; and the œdematous infiltrations not only fill all the exterior areolar tissues, but the serous cavities, and the pulmonary tissue, the latter often being the immediate cause of a fatal result. Cases of this form of cardiac disease vary greatly in the rapidity of their progress. Some will pass through all the stages I have described to you, reaching a fatal result in one or two years; while others will progress so slowly that the patients are able to transact business most of the time for ten or fifteen years, and do not reach a fatal result in twenty years.

*Prognosis:* After the detailed account I have given you concerning the nature and extent of the changes taking place in the class of cases under consideration, it is hardly necessary to add that the prognosis, so far as it relates to ultimate recovery, is always unfavorable. Yet very much can be done by judicious treatment, especially in such cases as are brought under the care of the physician early, both to mitigate the suffering of the patient and to prolong his life. So true is this, that no patient of this kind should be abandoned by his physician, merely because he cannot be actually cured; for the obligation to mitigate suffering and prolong life, is as binding upon the physician as is that of curing disease. And I may add, that in no class of incurable diseases can the judicious and skillful physician be of more real service to his patient, than in those involving the structure and functions of the heart.

*Treatment:* I need only remind you that in a primary attack of rheumatic endocarditis, the most efficient anti-rheumatic treatment should be promptly resorted to and continued, if possible, until all physical signs of cardiac trouble have ceased. But when called to cases like the one before you, in which the acute or primary attack has long since passed by, and the cardiac changes have become permanent, what are the indications for treatment, and what the means best calculated to fulfill those indications? If you have clearly perceived the nature and order of progress of the symptoms, as I have endeavored to describe them to you, there should be no difficulty in your recognizing two well defined indications to be constantly kept in view in this and all similar cases; namely, to counteract or overcome the rheumatic susceptibility by which the patient is made constantly liable to exacerbations from slight atmospheric changes, or other causes; and to keep the heart's action sufficiently slow to allow sufficient blood to pass through the obstructed auriculo-ventricular opening to fill the ventricle between the systoles.

In proportion as we can accomplish the first, we shall prevent or retard the further thickening of the valvular structure and the parts to which it is attached. And so long as we can maintain the second, we shall keep the patient free from dyspnœa and the whole train of evils resulting from the incomplete oxygenation and decarbonization of the blood.

The means best adapted for overcoming the rheumatic diathesis or morbid susceptibility of the structures, embrace both hygienic and remedial agents. For instance, the patient should have a warm alkaline bath every three or four days. When he comes out of the bath, the water should be wiped off quickly with ordinary towels, and the whole surface briskly rubbed with dry, soft flannel. This brings a pleasant glow of electric warmth to the surface, and renders the skin healthy and active.

Thick and warm underclothes of flannel should be worn all the year except July and August, during which months light canton-flannel may be substituted in place of the thicker cloth.

The air of the patient's apartments should be dry and well ventilated; his exercise moderate but habitual; his diet plain, nutritious, but not rich or highly seasoned; and his drinks unstimulating. To these may be added a protracted use of some medicines, with a reasonable expectation of benefit.

In these purely chronic cases I have, for many years, used different preparations of the cimicifuga, the phytolacca, and the stramonium, either singly or combined in various proportions, and with considerable benefit. Nearly two years since, my attention was called to the use of the senecio aureus as a remedy of value in relieving chronic rheumatic irritation in any of the fibrous structures of the body, and especially for removing that state of morbid sensitiveness we call the rheumatic diathesis. I had under treatment at the time a delicate girl, nine years of age, who had been attacked four years previously with severe acute rheumatism, that involved in its progress nearly all the articulations and the left side of the heart. After a protracted period of suffering, she recovered, leaving a well marked rough bellows murmur over the left cardiac region, with the usual embarrassment on taking active exercise. She had continued extremely sensitive to atmospheric changes, and had renewed attacks of rheumatic fever, with swelling and pain in some of the articulations, and increased cardiac disturbance, two or three times a year, in spite of the most vigilant precautions. She spent one of the winters in Florida, but had a renewal of the rheumatism in a few weeks after her return. It was at this time that a friend of the child's father told him, if he would get some senecio aureus root, put it into whisky, and give the girl some three times a day, it would cure her. The positive assurances of the friend evidently made an impression on the father's mind, and induced him to call my attention to the matter. On referring to such books as were at hand, I found the article named in the list of secondary articles of the Materia Medica, with no other account of its virtues than the statement that it was reputed to possess moderate diaphoretic and diuretic properties. Thinking that it would do no harm to gratify the father's desire to try the remedy, and finding no preparation of it in the drug-stores except a small quantity of fluid extract, the little patient was put upon the use of this, commencing with 0.3 C. C. three times a day. Subsequently some fresh root was procured, and a perfectly reliable fluid extract prepared for her use. The dose was gradually increased to 0.6 C. C., and its use was continued faithfully seven or eight months. No other remedies were used, and no changes were made, either in hygienic management or residence; but from the time she commenced to take the remedy, until the present time — nearly two years — she has not had the slightest return of rheumatic irritation, and has slowly increased in flesh and strength, and is now able to exercise quite freely with her playmates. There is still an audible murmur over the left side of the heart, but much less rough and harsh than two years ago. At no time during the use of the remedy has there been observable any active disturbance of the functions of the system. From the first, her appetite began to improve, and the functions of the skin, kidneys and bowels have been performed with entire regularity. Only once has there been need of medical interference, and that only for two or three days, on account of slight sore throat.

Of course the result in this case, standing alone, would prove nothing. The use of the remedy and the subsequent change in diathesis might be a mere coincidence. But during the past year or eighteen months I have prescribed the remedy in the form of fluid extract in a large number of cases, and with sufficiently favorable results to justify you and the profession generally, in making a thorough investigation concerning its value and remedial properties.

While on this subject, there is one important error concerning which I wish to caution you, and that is, the tendency, when treating chronic diseases, and especially when endeavoring to correct morbid diathesis or constitutional conditions, to change remedies too often. We are apt to forget that most of these conditions are the result of slowly acting causes, and involve alterations in the inherent properties of the tissues that can be changed back in the direction of health only by slow and persistent influences, both of a hygienic and medicinal character.

The senecio aureus is a plant that grows in sufficient quantities throughout the northern belt of the United States, and is familiar to medical botanists.

In fulfilling the second indication, namely, to keep the systolic action of the heart slow enough to allow the blood to pass fully from the left auricle into the ventricle, and thereby prevent too much fullness of the pulmonary veins, we must rely upon the judicious regulation of the patient's exercise, both mental and physical, and on the employment of such sedatives as will control the vascular excitement with the least tendency to impair the functions of digestion and assimilation. If possible, the mind of the patient should be occupied with some light, cheerful business, that does not involve intensity of application or anxiety ; and all his physical exercise should be light, habitually in the open air, but studiously avoiding all hurry or sudden violence. So long as the functions of respiration and digestion remain good, and the action of the heart steady, no special cardiac sedatives may be necessary. But whenever the obstruction is such that the action of the heart becomes habitually frequent or unsteady, and the breathing short and oppressed from slight exercise, a judicious use of digitalis will be of great value. For an adult, it is well to begin with doses of 1.3 C. C. of the ordinary tincture, or 0.8 C. C. of a good fluid extract, and repeat it every four hours until the pulse is reduced a little below the natural standard of frequency; then lengthen the interval between the doses, just enough to maintain the steady, slow beat, without further depression. The greater permanency of the action of the digitalis, and its less liability to disturb the stomach, make it preferable to most of the recognized arterial or cardiac sedatives, in this class of cases. In addition to these general indications, nearly all the cases that come under the observation of the physician require more or less collateral attention to the digestive organs and kidneys. You will remember that the patient before you suffers more or less from indigestion, characterized by acid and gaseous eructations and sometimes vomiting, with more or less constipation. To correct this, something that will act as an antiseptic and sedative to the irritability of the gastric mucous membrane is needed. I have found nothing that would fullfil these requirements better than small doses of carbolic acid and gelseminum, given just before each meal and at bed-time. Indeed, we may include nearly all the remedies indicated in the case before you, in the following prescription:

| | | | |
|---|---|---|---|
| ℞ | Acid carbolic (crystal)......... | 0 | 40 grams. |
| | Glycerine (pure)................. | 16 | 00 " |
| | Tinct. gelseminum ............ | 16 | 00 " |
| | Tinct. digitalis...... ........... | 32 | 00 " |
| | Fl. ext. senecio au............. | 96 | 00 " |
| | Mix. | | |

Give five grams, or an ordinary teaspoonful, in a little water, just before each meal and at bed-time. The steady use of this, with due attention to diet and exercise, and the avoidance of all use of alcoholic drinks and tobacco, will probably do as much to counteract the rheumatic diathesis, regulate the action of the heart, improve digestion, and thereby prolong the life and usefulness of the patient, as any course of treatment we could suggest.

